# Supplementary material for: Identification and functional analysis of non-coding regulatory small RNA FenSr3 in Bacillus amyloliquefaciens LPB-18
Source: PeerJ. 2023 May 15;11:e15236. doi: 10.7717/peerj.15236 (PMC10194069; doi:10.7717/peerj.15236)
Supplement: Supplemental Information 4 [file peerj-11-15236-s004.zip › KO/CK-vs-T1_map/map04664.html]

KEGG PATHWAY: Fc epsilon RI signaling pathway - Reference pathway


|  |  |
| --- | --- |
| **Fc epsilon RI signaling pathway - Reference pathway** |  |

[
Pathway menu
| Organism menu
| Pathway entry
| Show description
| User data mapping
]

Fc epsilon RI-mediated signaling pathways in mast cells are initiated by the interaction of antigen (Ag) with IgE bound to the extracellular domain of the alpha chain of Fc epsilon RI. The activation pathways are regulated both positively and negatively by the interactions of numerous signaling molecules. Mast cells that are thus activated release preformed granules which contain biogenic amines (especially histamines) and proteoglycans (especially heparin). The activation of phospholipase A2 causes the release of membrane lipids followed by development of lipid mediators such as leukotrienes (LTC4, LTD4 and LTE4) and prostaglandins (especially PDG2). There is also secretion of cytokines, the most important of which are TNF-alpha, IL-4 and IL-5. These mediators and cytokines contribute to inflammatory responses.

|  |  |  |
| --- | --- | --- |
| Reference pathway | 184% 150% 122% 100% 82% 67% 55% | 图片下载 |
